# Heterogeneity of neutrophils in cancer: one size does not fit all

**DOI:** 10.20892/j.issn.2095-3941.2022.0426

**Published:** 2022-12-12

**Authors:** Song Chen, Qingyu Zhang, Lisha Lu, Chunhui Xu, Jiajia Li, Jiali Zha, Fengxia Ma, Hongbo R. Luo, Alan Y. Hsu

**Affiliations:** 1 State Key Laboratory of Experimental Hematology, National Clinical Research Center for Blood Diseases, Haihe Laboratory of Cell Ecosystem, Institute of Hematology & Blood Diseases Hospital, Chinese Academy of Medical Sciences & Peking Union Medical College, Tianjin 300020, China; 2 Department of Pathology, Harvard Medical School, Boston, MA 02115, USA; 3 Department of Laboratory Medicine, The Stem Cell Program, Boston Children’s Hospital, Boston, MA 02115, USA; 4 Dana-Farber/Harvard Cancer Center, Boston, MA 02115, USA

**Keywords:** Neutrophils in cancer, tumor microenvironment, TAN, neutrophil heterogeneity, neutrophils

## Abstract

Neutrophils play an essential role in the defense against bacterial infections and orchestrate both the innate and adaptive immune responses. With their abundant numbers, diverse function and short life span, these cells are at the forefront of immune responses, and have gained attention in recent years because of their presence in tumor sites. Neutrophil involvement pertains to tumor cells’ ability to construct a suitable tumor microenvironment (TME) that accelerates their own growth and malignancy, by facilitating their interaction with surrounding cells through the circulatory and lymphatic systems, thereby influencing tumor development and progression. Studies have indicated both pro- and anti-tumor properties of infiltrating neutrophils. The TME can exploit neutrophil function, recruitment, and even production, thus resulting in pro-tumor properties of neutrophils, including promotion of genetic instability, tumor cell proliferation, angiogenesis and suppression of anti-tumor or inflammatory response. In contrast, neutrophils can mediate anti-tumor resistance by direct cytotoxicity to the tumor cells or by facilitating anti-tumor functions *via* crosstalk with T cells. Here, we summarize current knowledge regarding the effects of neutrophil heterogeneity under homeostatic and tumor conditions, including neutrophil phenotype and function, in cancer biology.

## Introduction

In humans and mice, 50%–70% and 10%–25% of circulating white blood cells are neutrophils, respectively^1,2^. This difference has been attributed to three factors: (1) evolutionary divergence between humans and mice over the course of approximately 10 million years, (2) different body sizes and lifespans, and (3) different microorganisms encountered in their respective environments^3–5^. Neutrophils are short-lived cells with an average half-life of 18.5 hours. Thus, to be constantly present in the circulation, they must be replenished in large quantities from the bone marrow (BM) through a process called granulopoiesis. In fact, the daily requirement is approximately 10^11^ neutrophils for humans and 10^7^ for mice^6,7^. These notable properties led to the early insight that neutrophils are professional effector cells that perform a particular set of functions in immune defense. As the first cells recruited to inflammation sites, neutrophils are the primary players in innate immunity, mediating inflammation and combating bacterial infection. Moreover, they facilitate the bridging and activation of adaptive immunity^1^. Neutrophils are also recruited to tumor sites in response to various stimuli and perform complex context-dependent functions within the tumor vicinity. The importance of neutrophils in regulating tumor development and metastasis has only begun to be explored in the past decade, but numerous advancements have been made, as discussed in the sections below.

The tumor microenvironment (TME) is a complex system that consists of tumor cells, tumor extracellular matrix, fibroblasts, blood vessels, and immune cells. The cells in the TME together promote tumor progression by producing various growth factors, cytokines, and chemokines^8^. The roles of neutrophils in the TME have not been as extensively explored as those of other myeloid cells, such as dendritic cells (DCs) and macrophages^9–11^. However, increasing evidence demonstrates that neutrophils are indispensable in the function of the TME and underscore their essential roles in promoting tumor growth and metastasis, as well as in the orchestration of pathways leading to tumor resiliency. However, neutrophils have also been shown to possess tumor killing and suppressive properties^12–14^. The intrinsic heterogeneity of neutrophils, in which their plasticity can be reshaped by the TME or other immune surroundings, underlies these contradictory findings. Advanced technologies, such as high-dimensional transcriptomic datasets and single-cell resolution cell profiling, can reveal neutrophil heterogeneity within healthy tissues and diseased conditions^15–18^. In the following sections, neutrophil heterogeneity in physiological and tumor conditions is comprehensively discussed. First, we describe recent high-resolution analyses of neutrophil differentiation in the physiological state. We then provide an overview of heterogeneous neutrophil populations in tumor conditions. Subsequently, we emphasize current understanding of the dual opposite roles of neutrophils and the mechanisms underlying how neutrophils participate in tumor growth and metastasis.

## Neutrophil differentiation, mobilization, and death

### Neutrophil differentiation and development

**Figure 1:**
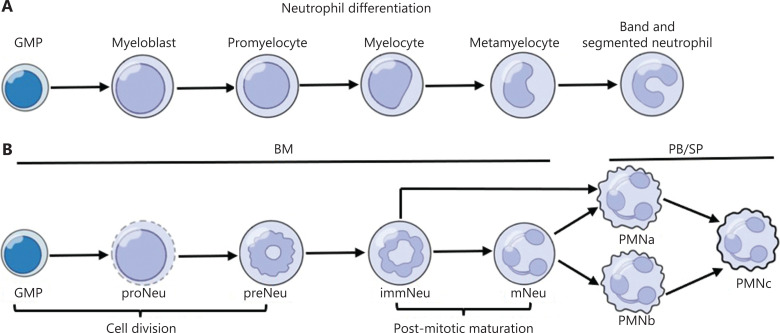
Neutrophil differentiation in steady state. (A) Traditional characterization of neutrophil development includes the following stages: GMP, myeloblast, promyelocyte, myelocyte, metamyelocyte, band and segmented neutrophil. (B) On the basis of trajectory analysis, the first 5 clusters, GMP, proNeu, preNeu, immNeu, and mNeu, originate primarily from the BM and represent neutrophil development in the BM. Neutrophil differentiation in BM is divided into 2 phases: cell division and post-mitotic maturation. The cell division stage is defined when GMP differentiate into proNeu and preNeu sequentially. The post-mitotic maturation stage encompasses immNeu, which give rise to non-proliferating mNeu. PMNa, PMNb, and PMNc are the major neutrophil subpopulations in the peripheral blood (PB) and spleen (SP). PMNa cells in the PB are derived from both mNeu and immNeu cells, whereas PMNb cells arise primarily from BM mNeu cells in the steady state.

In the BM, neutrophil development starts from granulocyte monocyte progenitors (GMP). After lineage commitment, neutrophil differentiation progresses through the following stages: myeloblasts, promyelocytes, myelocytes, metamyelocytes, band cells, and segmented nucleus neutrophils^19^ (**Figure 1A**). Different stages of neutrophil differentiation are usually classified by morphological features including cell size, nuclear condensation, cell marker expression, differentiation properties, and protein content^2^. However, classifying neutrophil differentiation on the basis of appearance is subjective and may not represent the entire differentiation process or correlate with function. Thus, recent reports have used cellular marker expression, single-cell RNA sequencing (scRNA-seq), and proteomics to better define the stages of neutrophil differentiation.

Improving transcriptomic analysis for single cells has enabled studies of hematopoietic dynamics, and separated neutrophil precursors into early, intermediate, and late phases with specific signature genes and transcription factors^20^. Additionally, studies using cytometry by time-of-flight (CyTOF) and cell-cycle-based analysis have revealed that neutrophil development in the BM has 5 stages: the earliest committed neutrophil progenitor (proNeu1) develops into intermediate progeny (proNeu2), which then progresses to a committed proliferative neutrophil precursor (preNeu), which undergoes further differentiation into non-proliferating immature neutrophils (immNeu) and subsequently mature neutrophils (mNeu)^21^. Xie et al.^16^ have shown that, on the basis of analysis of the BM, peripheral blood, and spleen Gr1^+^ cells with scRNA-seq, neutrophils can be separated into 8 major populations (G0–G4 and G5a–G5c) with distinct molecular signatures. According to their analyses, the G0, G1, G2, G3, and G4 clusters correspond to BM GMP, proNeu, preNeu, immNeu, and mNeu, respectively, whereas G5a–G5c have polymorphonuclear morphology typical of PMNa, PMNb, and PMNc, the most differentiated neutrophils (**Figure 1B**). By using CyTOF and scRNA-Seq methods, Zhu et al.^18^ have identified a continuum of clusters in Ly6G expressing cells composed of 2 major populations: C1 and C2. Comparison of the C1 and C2 cells with the neutrophil subpopulations identified by Xie et al.^16^ has indicated that C1 and C2 are the concoction of G0/G1 and G2/G3, respectively. Furthermore, Giladi et al.^15^ have separated developing neutrophils into stage I and stage II. Comparison with the 8 neutrophil populations from Xie et al.^16^ has revealed that stage I is primarily G2–G4 cells, and stage II is a blend of G4 and G5 cells. Recently, Grieshaber-Bouyer et al.^22^ have identified 4 neutrophil clusters (P1–P4) and demonstrated that P1 cells correspond to preNeu. P1–P3 are most plentiful in the BM, and P4 is most plentiful in the circulation and spleen, in agreement with findings from the abovementioned studies on the neutrophil maturation process. Calzetti et al.^17,23,24^ have identified CD66b^−^CD64^dim^CD115^−^ neutrophil-committed progenitor cells (NCPs) within the SSC^lo^CD45^dim^CD34^+^ and CD34^dim/−^ subsets of human BM and suggested that these NCPs are upstream of the previously described early neutrophil progenitors and proNeu, which generate human CD66b^+^ neutrophils exclusively. NCP clusters differ in the degree of maturation and eventually differentiate into 2 development routes, thus resulting in 4 subpopulations in total. These refined definitions of the development stages of neutrophils indicate that the differentiation of neutrophils is a complex and heterogeneous process.

### Neutrophil mobilization

The release of neutrophils from the BM into the circulation is a tightly regulated process controlled by signaling pathways of the chemokine receptors CXC-chemokine receptor4 (CXCR4) and CXCR2 located on neutrophil precursors^25^. In steady state, osteoblasts and other BM stromal cells express CXCL12, which signals CXCR4^+^ neutrophils to remain inside the BM. During neutrophil maturation, CXCR4 expression is gradually downregulated, and the BM is consequently unable to retain neutrophils and releases them into the circulation. Moreover, the expression of CXCR2 is elevated on mature neutrophils, thus facilitating neutrophil emigration into the circulation through interaction with CXCL1 and CXCL2 produced by endothelial cells and megakaryocytes^26^.

### Neutrophil aging and death

Neutrophils are discharged into the circulation according to circadian rhythms, and both circulating human and mouse neutrophils display different phenotypes throughout the day^2,27^, as evidenced by the fluctuating expression of CD62 L^+^ and CXCR2^+^ on newly released mature neutrophils in the circulation. Over time, in the circulation, neutrophils age and gradually lose expression of CD62 L, while CXCR4 and CD11b expression is upregulated, and the nuclei become hypersegmented^2,28,29^. These CXCR4^+^CD11b^+^ CD62 L^low^ aged neutrophils display enhanced activation of integrin and a remarkable ability to form neutrophil extracellular traps (NETs) in mouse venules during inflammation^30^. These phenotypic changes are caused by circadian cycling of the transcription factor brain and muscle Arnt-like protein 1 (BMAL1)^27,29^. BMAL1 upregulates the chemokine CXCL2, thus leading to CXCR2-induced diurnal changes in the transcription and migration of neutrophils in the circulation^27,29^. Aged neutrophils are believed to return to the BM or liver, and to be eliminated by macrophages at the end of their life cycle^28^. However, this mechanism alone cannot account for all neutrophil turnover. Therefore, knowledge regarding the destination of most aged neutrophils, and whether it relates to the uninterrupted replenishment of circulating neutrophils, remains lacking.

Both senescent neutrophils^31^ and neutrophils during infection^32^ can die in the vasculature or tissue and are then eliminated by Kupffer cells, a type of macrophages in the liver vasculature or other resident macrophages^32^. Most physiological neutrophils have been suggested to die *via* apoptosis, a non-inflammatory cell death. Previous studies have found that the removal of apoptotic neutrophils by both Kupffer cells and DCs appears to be controlled by regional interleukin-23 (IL-23), IL-17, and granulocyte colony-stimulating factor (G-CSF) signaling. The function of this cytokine axis promotes neutrophil maturation in the BM^33^ for replenishment and is down-regulated by liver X receptors^31^. Inflammatory lytic or violent cell death, such as pyroptosis, can be mediated in a caspase dependent or independent manner^34^. Necroptosis is a more lytic form of caspase-8 dependent programmed cell death that occurs when receptor interacting protein kinase-3 (RIPK3) and mixed lineage kinase domain-like protein (MLKL) pathways are triggered by bacteria such as *Staphylococcus aureus*^35^. Neutrophils may also die *via* several other lytic or inflammatory methods^36,37^. For instance, during NETosis, neutrophils burst and release their genetic matter to the extracellular space, releasing a web of DNA with toxic enzymes which can directly trap, contain, and kill microorganisms^38^. Neutrophils can also die *via* autophagy, necrosis, and phagocytosis^36,37^. Therefore, depending on the surrounding environment, different types of cell death occur, thus suggesting that neutrophil death involves a complex crosstalk between various intrinsic and extrinsic pathways.

## Neutrophil recruitment in tissues and tumors

After neutrophils enter the bloodstream, they are prepared for entry into target tissues in response to inflammatory or chemoattracting cues. Extravasation of neutrophils across the vessel wall is a five-step process involving tethering, rolling, adhesion, crawling and transmigration, and is tightly regulated by adhesion molecules expressed on vascular endothelial cells, which interacts with the integrins expressed on neutrophils^19^. Neutrophils begin extravasation *via* tethering on the endothelium surface and subsequently roll along the vessel. For neutrophils to remain attached, the neutrophil ligand P-selectin glycoprotein ligand 1 (PSGL1) interacts with the endothelial cells adhesion molecules P-selectin and E-selectin^39^, thus allowing neutrophils to migrate along the endothelial surface. In addition, L-selectin expressed on circulating neutrophils facilitates secondary tethering^40^ and strengthens adhesion to the endothelium, thus enabling transmigration into the tissue^39^. The firm adhesion of rolling neutrophils is regulated primarily by the engagement of integrin lymphocyte function-associated antigen 1 (LFA-1) and its ligands intercellular adhesion molecule 1 (ICAM1) and ICAM2, which are expressed on the endothelium. Once firmly bound on the vascular endothelium, neutrophils transmigrate from the circulation to the inflamed tissue, where they exert various functions according to the inflammatory context or tissue^41^.

After neutrophil recruitment to tumor and cancer sites^42^, many cytokines, growth factors, and chemokines, including Il-17, IL-1β, G-CSF, CXCL1, and CXCL2, which participate in the maturation and mobilization of neutrophils, are frequently increased in the TME^43,44^. The underlying reason is that tumor cells, tumor-associated stromal cells, and tumor infiltrated immune cells contribute to elevated production of these cytokines and chemokines in tumor-bearing mice^1^. Tumor-derived G-CSF mobilizes neutrophils and facilitates their homing to non-neighboring tissues, thus enabling influx of tumor cells^45^. Tumor-associated macrophages (TAMs) produce IL-1β^46^, which promotes T cell secretion of IL-17, which in turn enhances G-CSF levels in the blood and facilitates the mobilization of neutrophils into the circulation^47^. Abnormal regulation of cytokines and chemokines by tumors or tumor-associated immune cells can disrupt the homeostasis of neutrophil preservation in, and release from, the BM.

As described above, neutrophils have high expression of CXCR1 and CXCR2, both of which interact with their ligands secreted by tumor cells, stromal cells, endothelial and immune cells in the TME, thus promoting their recruitment to tumor sites^1,48,49^. In addition to chemokines, tumor cells and tumor-infiltrating immune cells have been reported to secrete inflammatory cytokines implicated in the same pro-tumor function of neutrophils^27,47,50^. Tumor necrosis factor-alpha (TNFα) secreted from T cells increases neutrophil recruitment to tumor sites, and together with the high IL-17 levels in the TME, leads to tumor-promoting actions of the recruited neutrophils^50^. The tumor-secreted protease cathepsin C (CTSC) activates proteinase 3 (PR3) on neutrophil membranes, thus aiding in IL-1β processing and activation of nuclear factor κB, and leading to enhanced expression of IL-6 and CCL3 recruiting neutrophils to tumor sites^51^. Therefore, neutrophil recruitment to the TME entails upstream regulation of granulopoiesis and a complex network of chemokines and cytokines that traffic neutrophils to tumor sites.

## Neutrophil heterogeneity

### Neutrophil heterogeneity in physiological and inflammatory conditions

Neutrophils in the bloodstream and organs show considerable heterogeneity in phenotypes and functions, owing to differences in parameters including maturation, aging, activation state, and signals sent by tissues^2,27^. Xie et al.^16^ have identified 3 major clusters (G5a–G5c) of neutrophils in the peripheral blood and spleen. G5a neutrophils have high expression of *Mmp8*, *S100a8*, and other migration and inflammatory response related genes. G5b neutrophils express high levels of interferon-stimulated genes, such as *Ifit3* and *Isg15*, thus facilitating interferon signaling, and the G5c subpopulation which had the highest aging score and therefore is suggested to be more aged or terminal than G5a and G5b. All 3 clusters are present during normal and inflamatory condition. Deerhake et al.^52^ have analyzed neutrophil heterogeneity in the lungs of mice infected with *Cryptococcus neoformans* (*Cn*), and identified 2 separate neutrophil populations in the lungs during acute *Cn* infection: one with an oxidative stress signature (Ox-PMN) and the other with elevated cytokine gene expression (Cyt-PMN)^52^. The authors have proposed that Ox-PMNs target the fungus and produce reactive oxygen species (ROS), whereas Cyt-PMNs have a longer lifespan, thereby allowing crosstalk with other immune cells such as DCs and alveolar macrophages, and facilitating a long term and specific anti-fungal response^52^. Thus, neutrophils carry distinct signatures which correspond to specialized functions, in conjunction with distinct differentiation routes together display heterogeneity in physiological and inflammatory conditions.

### Neutrophil heterogeneity in cancer

The TME polarizes TAMs toward a pro-tumor (M2) phenotype from an original anti-tumor (M1) phenotype^53^. Like TAMs, tumor-associated neutrophils (TANs) can differentially polarize, thus resulting in a similar classification standard: TANs can be classified into an anti-tumorigenic (“N1”) or pro-tumorigenic (“N2”) phenotype^54^. TGF-β is an abundant and important cytokine in the vicinity of tumors, which induces neutrophils to adopt a N2 TAN phenotype. In contrast, blocking TGF-β in the TME induces a functional switch in associated neutrophils to an anti-tumoral phenotype^54^. N1 TANs have efficient tumor cell killing capability and high expression of immune activation related cytokines and chemokines, including CCL3, CXCL9, and CXCL10, which promote the recruitment and activation of CD8^+^ T cells. In contrast, N2 TANs promote pro-tumor function by producing large amounts of arginase 1 (Arg1)^55^, which downregulates the surrounding level of L-arginine, thus leading to T cell dysfunction and hindering anti-tumor responses mediated by T cells^54^. In summary, this framework of classifying TANs distinguishes their dual roles in tumor progression and explains the apparently contradictory findings from studies on neutrophil functions in cancer^54^.

The heterogeneity of circulating neutrophils can also be characterized according to sedimentation proprieties into high density neutrophil (HDNs) and low-density neutrophils (LDNs) under tumor conditions. HDNs consist of mature neutrophils, which are predominant in healthy individuals and have cytotoxic abilities toward tumor cells, whereas LDNs increase during tumor progression and have impaired neutrophil functions, immunosuppressive properties, and other characteristics opposite from those of mature HDNs. LDNs consist of 2 types of cells: immature myeloid-derived suppressor cells (MDSCs) and original HDNs, whose functional properties are converted by TGF-β-dependent signaling in the tumor mass^56,57^.

Single cell transcriptomic advances have permitted detailed characterization of the heterogeneity of neutrophils in the TME. ScRNA-seq data for splenic neutrophils and monocytes from a breast cancer mouse model have been compared with those for wild-type controls, and revealed that neutrophils form a continuum of phenotypes consisting of various unique clusters (C0, C2, C4, C5, C7, and C8). C0 is defined by high expression of genes, such as Camp*17*, indicative of mature neutrophils and high *Ly6g* expression; C2 significantly increases in mice carrying tumors and also has notable expression of immune regulating genes such as *Il1β* and *Arg2*; C4 and C5 overlap in marker genes including *Cebpe* and *Retnlg*; and C7 and C8 uniquely have high expression of cell cycle genes such as *Tuba1b* and *Cdc20*, thus suggesting existence of a neutrophil reservoir in the spleen, where neutrophils can be replenished^58^. ScRNA-seq analysis of infiltrating myeloid cells in human and mouse lung tumors has revealed that neutrophils establish a plethora of phenotypic states comprising 5 (hN1–hN5) cell subsets in humans and 6 (mN1–mN6) in mice. h/mN1 neutrophils have high expression of canonical neutrophil markers such as *MMP8*, *MMP9*, *S100A8*, *S100A9*, and *ADAM8*; h/mN2 neutrophils express high levels of interferon response genes such as *IFIT1*, *IRF7*, and *RSAD2*; hN3 and hN4 express high levels of *CASS4* and *CTSC*, respectively; mN3 and mN4 express *Cxcl3* and *Pald1*, respectively; h/mN5 produces the cytokines *CCL3* and *CSF1*, as well as *CSTB*, *CTSB*, and *IRAK2*; and mN6 uniquely expresses *Fcnb* and *Ngp*. Moreover, 3 neutrophil subsets (mN4–6) have been identified, expressing the sialic acid binding Ig-like lectin F (SiglecF). Thus, the 6 subpopulations can be divided into 2 categories: SiglecF^hi^ neutrophils, which are pro-tumor and contribute to the TME, and SiglecF^lo^ neutrophils (mN1–3), which exist in healthy non-tumor lungs^59^.

On a cell surface marker level, CyTOF plus high-dimensional analysis of blood samples from melanoma-bearing patients without treatment has indicated that neutrophils in the blood cluster into 7 populations: the CD117^+^CD66b^+^CD38^+^ neutrophil progenitor hNep; the subpopulation of CD16^dim^CD62 L^bright^ band cells Cneut1; the terminally differentiated mature neutrophils Cneut2, with high expression of CD101, CD10, CD16, and CXCR2; the CXCR4^+^CD49d^+^CD62 L^lo^ aged neutrophils; Cneut3, the only cluster expressing CD45RA^+^; Cneut4, which produces the lowest level of ROS among all subsets; the immature neutrophils Cneut5; and finally Cneut6, another subpopulation of band cells that express exclusively CD14^+^. Furthermore, the hNeP and Cneut1 populations display the strongest phagocytic efficiency in all clusters. The Cneut2 and Cneut5 populations show the most robust ROS producing ability when stimulated, whereas Cneut6 and Cneut4 have lower ROS production capability. More than 95% of neutrophils from healthy donors were Cneut2, whereas all other subsets combined composed ∼5%. The proportion of Cneut2 decreased to < 90% while the other clusters increased to greater than 10% of total neutrophils, as determined by flow cytometry, in patients with melanoma. Furthermore, Cneut2 and Cneut5 became the most prevalent neutrophil subsets in the blood of patients with melanoma. Investigation of the 7 clusters has revealed that during tumor progression, neutrophil heterogeneity in the blood is altered in response to tumor signaling^60^.

To classify PMNs in a mouse tumor model, Veglia et al.^61^ have described 3 subpopulations of PMNs from tumor bearing mice: the classical PMNs, which account for almost 95% of neutrophils in control spleen; the polymorphonuclear myeloid-derived suppressor cells (PMN-MDSCs), which include immature neutrophils expressing PMN-MDSC-associated genes such as *Ngp*, *Ltf*, *Cd177*, *Anxa1*, *Mmp8*, *S100a8*, *S100a9*, *Cebpe*, *Ltb4r1*, and *Cybb*; and the activated, potentially immune suppressive PMN-MDSCs characterized by chemokines, chemokine receptors, and genes involved in cell activation, inflammation, and ER stress (*Ccl4*, *Ccl3*, *Cxcl2*, *Cxcl3*, *Spp1*, *Il1b*, *Nfkbia*, *Socs3*, *Mif*, *Klf6*, *Atf3*, *Ptgs2*, and *Xbp1*). The level of CD14 has been used to distinguish the 3 populations, given that CD14^hi^ neutrophils correlate with higher immunosuppressive gene expression.

Wang et al.^62^ have analyzed scRNA-seq metadata of human PMNs from peripheral blood and tumor-infiltrating leukocytes in 5 patients with pancreatic ductal adenocarcinoma, and identified 6 populations of TANs including the non-cluster-specific TAN-0; the terminally differentiated pro-tumor subpopulation TAN-1, associated with poor prognosis; the inflammatory subpopulation TAN-2; the TAN-3 subpopulation newly migrated to the TME; the TAN-4 subpopulation, which preferentially expresses interferon-stimulated genes; and an undefined TAN-5 subpopulation. In addition, recent studies have used a high-resolution single-cell atlas to analyze non-small cell lung cancer infiltrating neutrophil population profiles. Four TAN subsets (TAN-1 to 4) and 2 normal adjacent tissue-associated neutrophils subsets (NAN-1 and NAN-2) have been identified, and extensive heterogeneity in phenotypes has been observed across subpopulations^63^. The TAN-1 cluster expresses high levels of *CXCL1*, *CXCL2*, *CXCL8*, *ICAM1*, and *CD44*, which regulate the activation, recruitment, and cell adhesion of neutrophils, and the formation of NETs; the TAN-2 cluster, which highly expresses MHC II-associated genes such as *HLA-DRA*, *CD74*, and *HLA-DPB1*, and therefore may have immunogenic antigen presenting functions and exhibit anti-tumor immunity; and the TAN-3 cluster, which exhibits pro-tumor characteristics and high expression of the lipid metabolism-associated genes *PLIN2* and PLPP3, which promote tumor proliferation. TAN-3 also has high expression of *PLAU*, which encodes the plasminogen-activator urokinase (uPA), which activates extracellular matrix degrading proteases, and mediates tumor cell adhesion and migration by interacting with its cognate receptor, uPAR, expressed on tumor cells. Therefore, TAN-3 may play important roles in cancer cell proliferation and migration. Finally, the TAN-4 cluster highly expresses ribosomal-associated genes such as *RPL10*, *RPS2*, *RPS18*, and *RPL3*, thus suggesting an alterable transition to tumor endothelial cells. Additionally, the NAN-1 cluster highly expresses the alarmin *S100A12*, which mediates neutrophil proinflammatory functions; the NETosis co-factor *PADI4*; and the proangiogenic genes *PROK2* and *MMP9*. The NAN-1 and NAN-2 clusters are similar, but NAN-2 has lower expression of *S100A12, MME, and PROK2*. These findings suggest that anti-tumoral and pro-tumoral neutrophils can co-exist in a given tumor isolate.

Collectively, neutrophils have different subpopulations with diverse properties in the spleen, blood, lung, and tumor tissues, thus highlighting the phenotypic and functional heterogeneity in tumor conditions. The nomenclature of neutrophil heterogeneity is complex, because each definition is defined on the basis of a certain limited dataset or model. Thus, these classifications must be compared and unified into a standardized naming system, given that the same “type” of TAN might have different names across reports or classification systems. We have summarized the current classifications in **Table 1**. However, a comprehensive analysis of current datasets is needed to unify the terminology of this complex classification system.

**Table 1:** Neutrophil heterogeneity in cancer

Species	Tumor	Populations	Signatures and/or functions	Reference
Mouse	AB12, LKR, and TC1	N1 and N2	N1: anti-tumor; N2: pro-tumor	^54^
	4T1	LDN and HDN	HDNs: cytotoxic capability toward tumor cells; LDNs: impaired neutrophil function and immunosuppressive properties	^57^
	MMTV-PyMT transgenic mouse model of breast cancer	C0, C2, C4, C5, C7, and C8	C0: *Camp17* and *Ly6g*; C2: *Il1β* and *Arg2*; C4, C5: *Cebpe* and *Retnlg*; C7, C8: *Tuba1b* and *Cdc20*.	^58^
	LLC	Classical PMNs, PMN-MDSCs, and activated PMN-MDSCs	Classical PMNs: account for almost 95% neutrophils in control spleen; PMN-MDSCs: *Ngp*, *Ltf*, *Cd177*, *Anxa1*, *Mmp8*, *S100a8*, *S100a9*, *Cebpe*, *Ltb4r1*, and *Cybb*; activated PMN-MDSCs: *Ccl4*, *Ccl3*, *Cxcl2*, *Cxcl3*, *Spp1*, *Il1b*, *Nfkbia*, *Socs3*, *Mif*, *Klf6*, *Atf3*, *Ptgs2*, and *Xbp1.* potent immune suppressive activity	^61^
	KP1.9	mN1–mN6	mN1: *Mmp8*, *Mmp 9*, *S100a8*, *S100a 9*, and *Adam8*; mN2: *Ifit1*, *Irf7*, and *Rsad2*; mN3: *Cxcl3*; mN4: *Pald1*; mN5: *Ccl3*, *Csf1*, *Cstb*, and *Irak2*; mN6: *Fcnb* and *Ngp*	^59^
Human	NSCLC	hN1–hN5	hN1: *MMP8*, *MMP9*, *S100A8*, *S100A 9*, and *ADAM8*; hN2: *IFIT1*, *IRF7*, and *RSAD2*; hN3: *CASS4*; hN4: *CTSC*; hN5: *CCL3*, *CSF1*, *CTSB*, and *IRAK2*	^59^
	Melanoma	hNeP and Cneut1–Cneut5	hNep: CD117^+^CD66b^+^CD38^+^ neutrophil progenitors; Cneut1: CD16^dim^CD62 L^bright^ band cell; Cneut2: terminally differentiated, mature neutrophils; Cneut3: CXCR4^+^CD49d^+^	^60^
			CD62 L^lo^ aged neutrophils; Cneut4: no specific features; Cneut5: immature neutrophils; Cneut6: CD16^dim^CD62 L^bright^ band cells	
	Pancreatic ductal adenocarcinoma	TNA0–TNN5	TAN-0: no cluster-specific distinctive features; TAN-1: terminally differentiated pro-tumor subpopulation; TAN-2: inflammatory subpopulation; TAN-3: transitional stage subpopulation; TAN-4: expression of interferon-stimulated genes; TAN-5: undefined subpopulation of low-quality cells	^62^
	NSCLC	TAN1–4/ NAN1–2	TAN1: *CXCL8*, *CXCL1*, *CXCL2*, *ICAM1*, and *CD44*; TAN2: *HLA-DRA*, *CD74*, and *HLA-DPB1*; TAN3: *PLIN2*, *PLPP*, *MAP1*, *LC3B*, and *PLAU*; TAN4: *RPL10*, *RPS2*, *RPS18*, *RPL3.* NAN1: *S100A12*, *PAD14*, *PROK2*, and *MMP9*; NAN2: similar to NAN1 cluster, and decreased expression of *S100A12*, *MME*, and *PROK2*	^63^

## Pro-tumoral neutrophil functions

Neutrophils have been suggested to facilitate tumor growth and malignancy, as evidenced by research indicating that Csf3^-/-^ neutropenic mice present a modest tumor growth delay in an LLC1 tumor model and diminished urethane-induced lung carcinogenesis^12^. Mice implanted with B16 melanoma and treated with antibodies against Gr1 or G-CSF to deplete neutrophils show diminished myeloid cell infiltration, tumor growth, and angiogenesis^13,64^. These studies have demonstrated that neutrophils are crucial for fostering carcinogenesis. In this section, we discuss the pro-tumor effects of neutrophils through promoting DNA instability, tumor cell proliferation, angiogenesis, and inhibition of innate and adaptive immune responses.

### Neutrophils induce DNA damage, thus leading to cancer occurrence

**Figure 2:**
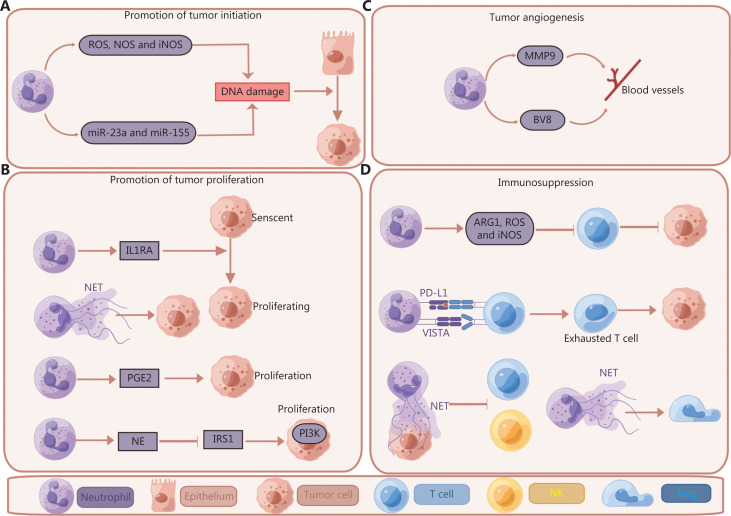
Neutrophils have protumor properties including causing DNA damage, and promoting tumor proliferation, angiogenesis, and immunosuppression. (A) Neutrophils cause DNA instability through genotoxic DNA substances including ROS, NOS, iNOS, and microRNAs such as miR-23a and miR-155, thus leading to tumor initiation and progression in multiple models (see text). (B) In a PTEN null prostate tumor model, neutrophils promote the proliferation of cancer cells by counteracting senescence with IL1RA production; anaplastic thyroid cancer conditioned medium induces TANs to release NETs in a mitochondrial DNA dependent manner, thereby promoting tumor proliferation. In a RAS-derived neoplasia zebrafish model, neutrophils produce PGE2, thus promoting proliferation of the pre-neoplastic cells in wounded tail fins. In a RAS-induced lung cancer model, neutrophil NE directly induces tumor cell proliferation by infiltrating into the endosomal compartment, degrading IRS-1, and activating PI3K signaling within tumor cells. (C) Neutrophils facilitate tumor angiogenesis *via* releasing MMP9 and BV8 in a transgenic mouse model with pancreatic islet cell carcinogenesis. (D) Immunosuppressive functions of neutrophils. Neutrophils produce Arg1, ROS, and iNOS, thus impairing the T cell-mediated anti-tumor response. In human hepatocellular carcinomas and gastric cancers, PD-L1^+^ neutrophils hinder the proliferation and activation of T cells, thus leading to the proliferation and progression of cancer cells. In a melanoma mouse model, VISTA expressed on neutrophils negatively regulates T cell-mediated antitumor immunity; NETs released in the TME form a protective layer on tumor cells and shield them from the cytotoxic activity of CD8^+^ T cells and NK cells. In a non-alcoholic steatohepatitis-hepatocellular carcinoma (NASH-HCC) model, tumor-induced NETs positively correlate with promotion of Treg differentiation in cancer by metabolic reprogramming of naïve CD4^+^ T-cells, thereby bolstering hepatocarcinogenesis.

The direct procarcinogenic effect of neutrophils can be induced by the production and release of genotoxic DNA substances that exacerbate DNA instability^65^. Chronic colon inflammation in patients triggers neutrophils to release genotoxic factors such as ROS and chlorinating agents (HOCl), which cause G2/M checkpoint arrest and replication errors in colon epithelial cells^66^. Neutrophil-derived production of ROS has also been demonstrated to induce oxidative DNA damage in tissues such as the lungs and intestines, thereby increasing the mutational load and eventually leading to cancer development and progression^67^. A lung chemical carcinogenesis model has shown that ROS produced from neutrophils induced DNA damage in the lungs, thus promoting tumor formation under carcinogen co-treatment^68^. In a subcutaneous cancer mouse model, the inducible nitric oxide synthase (iNOS) and nitric oxide synthase (NOS) content produced by infiltrating neutrophils closely correlates with the number of hypoxanthine phosphoribosyl transferase (Hprt) mutations, which contribute to the burden of genetic abnormalities associated with tumor progression^69^. In inflammatory bowel disease, activated neutrophils in tissues release microparticles containing proinflammatory microRNAs, including miR-23a and miR-155, which lead to DNA double-strand breaks and consequently genomic instability in a ROS-independent manner. Thus, during inflammation, impaired tissue healing and higher mutation rates due to genomic instability in the epithelium result in tumor initiation and progression^70^ (**Figure 2A**).

### Neutrophils promote tumor cell proliferation

Increasing evidence suggests that neutrophils play an important role in promoting tumor cell proliferation. For instance, in a RAS-derived neoplasia zebrafish model, neutrophils produce the trophic signal molecule prostaglandin E2 (PGE2), which increases the amount of pre-neoplastic cells in wounded tail fins^71^. Likewise, in RAS-induced lung cancer, neutrophil elastase (NE) has been found to directly cause tumor cell proliferation by infiltrating into endosomal compartments, degrading insulin receptor substrate-1 (IRS-1), and activating phosphoinositide 3-kinase (PI3K) signaling in tumor cells^72^. Furthermore, neutrophils have been found to counteract oncogene-induced senescence through inducing the expression of interleukin-1 receptor antagonist (IL-1RA) on tumors^73^. In anaplastic thyroid cancer, altering oxidative mitochondrial metabolism has been found to allow neutrophils to retain functionality and release NETs, thus promoting cancer cell proliferation^74^. Neutrophils can directly or indirectly lead to the proliferation of tumor cells (**Figure 2B**).

### Neutrophils promote angiogenesis

Neutrophils have an important role in enhancing angiogenesis by producing matrix metalloprotease type 9 (MMP-9), vascular endothelial growth factor (VEGF), and (prokineticin 2) BV8^75–77^. Tissue-infiltrating neutrophils are a major source of MMP9 secretion *in vivo* which can induce angiogenesis in the TME. Specifically, MMP-9 released by neutrophil tertiary granules^78,79^ breaks down the extracellular matrix and is followed by release of VEGF and increased angiogenesis^75^. In human breast cancer, neutrophils have also been found to produce cytokines, such as Oncostatin M, from the IL-6 family, which promotes the production of VEGF in cancer cells and increases angiogenesis and breast cancer cell detachment, thus aggravating invasive capacity^80^. In a transgenic mouse model with pancreatic islet cell carcinogenesis, VEGF-independent tumor angiogenesis is facilitated by Bv8 expressed by CD11b^+^Gr1^+^ neutrophils^77^ (**Figure 2C**). In summary, studies have revealed an essential role of neutrophils in angiogenesis during tumor initiation.

### Immunosuppression

Neutrophils produce ROS, iNOS, Arg1, prostaglandins, and ligands of immune checkpoint inhibitory receptors, thereby suppressing innate and adaptive lymphoid cell functions^1,7,64^.

Neutrophil-derived ROS have been widely acknowledged to be key inhibitors of T cell activation during cancer, particularly in advanced tumors^27^. In a 4T1 breast tumor mouse model, tumor-induced oxidative neutrophils produce ROS, thus decreasing T cell activation even when glucose utilization is limited by the competitive inhibitor 2-deoxy-D-glucose^81^. Neutrophils can also hinder T cell anti-tumor activity by producing NO *via* iNOS, as demonstrated in a tumor-bearing KEP mouse model^47^. Neutrophil derived Arg1 has been observed to block T cell mediated anti-tumor cytotoxicity through depleting L-arginine in both mouse and human cancers^54,82^. In addition, TGFβ from tumors enhances Arg1 production by neutrophils^54^. In human hepatocellular carcinomas, the PD-L1^+^ neutrophils from patients effectively hinder T cell proliferation and activation through interacting with the ligand PD-1, but this process is partially prevented by blocking PD-L1. In human gastric cancer, GM-CSF expressed by tumors induces the activation of neutrophils and triggers the expression of PD-L1 through activation of the Janus kinase (JAK)-signal transducer and activator of transcription 3 (STAT3) signaling pathways. Furthermore, PD-L1^+^ neutrophils efficiently inhibit T cell activation and promote the proliferation and progression of human gastric cancer *in vitro* and *in vivo*, respectively^83,84^. V-Domain Ig Suppressor of T Cell Activation (VISTA) is expressed on several immune cells, including CD11b^+^ myeloid cells, naïve CD4^+^ and CD8^+^ T cells, CD4^+^ Foxp3^+^ Treg cells, and TCRγδ T cells, and negatively regulates T cell-mediated autoimmunity and antitumor immunity^85^. In a study using a transplantation mouse model with melanoma, VISTA has been found to enhance the effector functions of MDSCs and tolerogenic DC subsets. VISTA blockade increases the production and release of proinflammatory mediators and decreases their T cell-suppressive functions^85^.

Immunosuppressive neutrophils associated with tumors are generally termed MDSCs. MDSCs were first introduced in 2007 as a subpopulation of myeloid cells characterized by expression of CD11b and Gr1, and having an immature myeloid state. Their ability to restrain T cell, B cell, and natural killer (NK) cell functions renders them immunosuppressive—a characteristic enriched in tumor scenarios but rare in homeostatic conditions^86,87^. PMN-MDSCs preferentially use ROS, peroxynitrite, Arg1, and PGE2 to mediate immune suppression^88^. Indeed, PMN-MDSCs and normal neutrophils are difficult to distinguish because they share the same set of markers and identical morphology, thus leading to confusion^89^. Although PMN-MDSCs are often ascribed immature characteristics to distinguish them from fully differentiated neutrophils^90^, Gr1 and Ly6G are expressed on both mature and immature neutrophils, thus hindering the use of cell markers to separate mature neutrophils from their progenitors. Moreover, all CD11b^+^Gr1^+^ cells in mice with tumors were once believed to be MDSCs, but in fact not all CD11b^+^Gr1^+^ cells are immunosuppressive^91^. Evidence from an epithelial ovarian cancer mouse model has indicated that CD11b^+^Gr-1^+^ myeloid cells in the ascites are immunostimulatory rather than being immunosuppressive during advanced stages of cancer. Those myeloid cells constitute a population displaying homogeneity and morphological resemblance to neutrophils. Furthermore, like DCs, CD11b^+^Gr-1^+^ cells that are immunostimulatory can efficiently cross-prime cytotoxic T lymphocytes and suppress tumor progression in a subcutaneous injection tumor-bearing mouse model through adoptive transfer^91^. Hence, PMN-MDSCs should be classified as a subset of TANs with immunosuppressive properties based on established definitions. However, further elucidation is warranted.

In addition, previous studies have demonstrated that NETs influence lymphocyte cytotoxicity in primary tumors. *In vivo* and *in vitro* experiments have demonstrated that NETs organize into a protective layer around tumor cells, which physically blocks the cytotoxicity of CD8^+^ T cells and NK cells^92^. In non-alcoholic steatohepatitis-hepatocellular carcinoma (NASH-HCC), regulatory T-cells (Tregs) bolster hepatocarcinogenesis by impeding Th1-mediated cancer immunosurveillance and blocking tumor-infiltrating CD8^+^ T cells. Furthermore, NASH-induced NETs have been shown to positively correlate with promotion of Treg differentiation in cancer through metabolic reprogramming of naïve CD4^+^ T-cells, thus linking innate and adaptive immunity in the liver in NASH, and indicating that the metabolic changes induced by the pro-tumoral environment also have a critical role in altering immunity^93^ (**Figure 2D**).

## Neutrophil antitumoral functions

Although most studies support that neutrophils are essential in promoting tumor progression, increasing evidence suggests that antitumoral neutrophil functions also exist. Previous studies have shown that neutrophils induce cell debridement^14^ and ROS-mediated cytotoxicity of tumor cells^94,95^, and proteases released from neutrophils have direct toxic effects on tumor cells^96–98^ and can directly eliminate them. Neutrophil-mediated killing of tumor cells can also occur through TNF-associated apoptosis inducing ligand (TRAIL) expression, and the release of Arg1 and tumor-derived TNFα^99–101^. A recent study has termed neutrophil-mediated cytotoxic activity, in which neutrophils mechanically destroy cancer cell plasma membranes and cause tumor cell death, “trogoptosis”^100,102^. In addition, neutrophils can indirectly crosstalk with T cells and facilitate tumor cell killing^103–106^. Therefore, in this section, we summarize the mechanisms underlying how neutrophils directly and indirectly kill tumor cells during tumor initiation and growth.

### Direct tumor cytotoxicity by neutrophils

Neutrophils directly terminate tumor cells through cell-contact dependent mechanisms and the production of ROS^14,107^. In a PTEN-deficient uterine cancer mouse model, neutrophil-induced debridement of tumor cells, in certain situations, eliminates the cells in nascent tumors and thus prevents uterine epithelial carcinogenesis^14^. ROS-mediated killing is achieved *via* the transient receptor potential cation channel, subfamily M, member 2 (TRPM2), which elicits influx of a fatal amount of Ca^2+^ into target cells in an H_2_O_2_-dependent manner^94^. TRPM2 expression is increased in tumor cells undergoing epithelial-to-mesenchymal transition, thus leading to increased production of CXCL2 by tumor cells and neutrophil recruitment to tumor sites. Therefore, besides initiating an apoptotic cascade in the tumor, TRPM2 supports neutrophil recruitment to tumor sites^94,95,97^. Consequently, TRPM2 has 2 distinct anti-tumor functions: promoting neutrophil recruitment to tumor sites and activating the apoptotic cascade in the tumor cells themselves.

**Figure 3:**
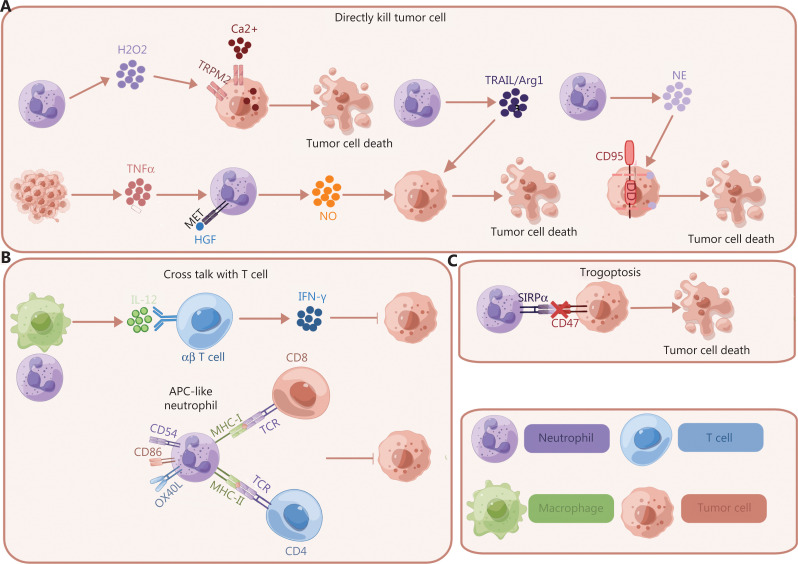
Neutrophils have direct and indirect antitumor effects. (A) Neutrophils directly kill tumor cells through the production of H_2_O_2_, in a process involving TRPM2, an H_2_O_2_-dependent channel, thus causing lethal influx of Ca2^+^ into breast cancer cells. In Lewis lung carcinoma and fibrosarcoma transplantation mouse models, tumor-derived TNFα induces MET expression on neutrophils, which then interact with HGF and promote the production and release of NO by neutrophils, thus killing tumor cells. In cytotoxic assays of neutrophils against the Jurkat human T cell leukemic cell line, neutrophils express TRAIL on the cell surfaces and release it into the culture medium, thus increasing leukemia cell apoptosis. Arg1 derived from activated neutrophils or dead cells has been shown to induce apoptosis of the HeLa human cervical epithelial carcinoma cell line as well as the SF268 human glioblastoma cell line through activation of the ER stress pathway. Neutrophils kill cancer cells and attenuate tumorigenesis through releasing catalytically active NE, which proteolytically liberates the CD95 death domain (DD) and leads to breast cancer cell apoptosis. (B) In 3-methylcholathrene (3-MCA)-induced sarcomagenesis, neutrophils increase the production and release of IL-12 in macrophages, thereby promoting the polarization and IFN-γ production in a subset of CD4^-^CD8^-^TCRβ^+^ unconventional T cells and exerting an anti-tumor response. Cross-talk between N1 TANs and activated CD4^+^ and CD8^+^ T cells induces the expression of costimulatory molecules such as CD54, CD86, OX40 L, and 4-1BBL on the neutrophil surface, which in turn promote T cell activation and INF-γ production. (C) Neutrophils directly kill breast cancer target cells *via* Fc-mediated destruction of the cancer cell plasma membrane (trogoptosis). Destruction of antibody-opsonized cancer cells mediated by neutrophils is enhanced by blocking the CD47-SIRPα do not-eat-me checkpoint.

Neutrophil-induced death of cancer cells can also be triggered by TRAIL expression and Arg1 release^99,100^. In cytotoxic assays of neutrophils against the Jurkat human T cell leukemic cell line, neutrophils produce TRAILs and subsequently release them into the surrounding area, thus increasing apoptosis of tumor cells^99^. Arg1 derived from activated neutrophils or dead cells has been shown to induce ER stress in tumor cells, thereby leading to their apoptosis^101^. In Lewis lung carcinoma and fibrosarcoma transplantation mouse models, tumor cell-derived TNFα prompts neutrophils to express the hepatocyte growth factor receptor (HGFR; also known as MET). The interaction between MET and HGF in the TME promotes the recruitment of neutrophils and the production of NO, thereby leading to tumor cell death^100^. However, as seen in a transplantation melanoma mouse model, HGF-MET signaling in neutrophils also elicits an immunosuppressive phenotype that contributes to impaired mobilization of antitumor T cells and is characterized by diminished effectiveness of T cell adoptive transfer responses to immune checkpoint blockade therapies^108^. These findings suggest the contextual dependence of neutrophil involvement. Therefore, how MET expression affects neutrophil functions remains to be thoroughly explored in diverse tumor contexts (**Figure 3A**).

Increasing evidence indicates that neutrophil proteins have an essential role in antitumoral function. Recent studies have shown that neutrophils discharge catalytically active NE, thus directly killing cancer cells, and attenuating tumorigenesis through proteolytically freeing the CD95 death domain in tumor cells, which in turn interacts with histone H1 isoforms and selectively leads to cancer cell apoptosis^96,109^. In glioblastoma, neutrophils transfer myeloperoxidase (MPO)-containing granules into tumor cells, and consequently facilitate iron-dependent aggregation of lipid peroxides inside tumor cells. Either inhibiting or removing MPO restrains neutrophil-elicited tumor cell cytotoxicity^97^. Although purified lactoferrin has been suggested to have anti-tumor properties^98^, its role in the context of tumor interaction *in vivo* has not been fully explored.

### Neutrophils indirectly induce tumor cell death via crosstalk with T cells

Neutrophils also elicit antitumor activities indirectly *via* crosstalk with T cells in the TME. During 3-methylcholathrene-induced sarcomagenesis, neutrophils enhance the secretion of IL-12 in macrophages. Consequently, IL-12 promotes polarization and interferon-γ (IFN-γ) production in a subset of CD4^-^CD8^-^TCRβ^+^ unconventional T cells, thereby initiating an anti-tumor immune response leading to tumor cell death^103^. Crosstalk between N1 TANs and activated T cells induces the expression of costimulatory molecules such as CD54, CD86, OX40 L, and 4-1BBL on neutrophil surfaces, thus promoting T cell activation and INF-γ production^104^. In addition, neutrophils have been shown to acquire an antigen presenting cell (APC) phenotype in early stage human lung cancers. These pseudo-APC “hybrid neutrophils” originate from immature CD11b^+^CD15^hi^CD10^–^CD16^int/low^ cells, and promote the proliferation and activation of both CD4^+^ and CD8^+^ T cells^104,105^. Neutrophils have also been found to secrete chemokines such as CCL2, CCL3, CXCL1, CXCL2, and CXCL10, thus directly mediating and promoting the recruitment of T cells as well as other leukocytes^106^ (**Figure 3B**).

### Neutrophils mediate tumor cell death via trogoptosis

Therapeutic antibodies mechanically destroy cancer cell plasma membranes at least partially through antibody-dependent cellular cytotoxicity (ADCC) by immune cells expressing Fc receptors, such as macrophages, NK cells, and neutrophils. The major routes through which NK cells and macrophages induce ADCC involve granule-dependent apoptotic and phagocytic mechanisms^110,111^. Neutrophils can be activated to destroy cancer cells *via* antibody-mediated cytotoxic activity, which is termed trogoptosis. Trogoptosis describes active and mechanical damage to cancer cell membrane integrity, thereby inducing a lytic and inflammatory type of cancer cell death. ADCC induced by neutrophils is unaffected by granule release and NADPH oxidase, and therefore differs from apoptotic pathways induced by NK cells, CTLs, and macrophage-mediated phagocytic cell death. Thus, trogoptosis is an anti-cancer cell destruction pathway used specifically by neutrophils and is not dependent on factors in the classical antimicrobial mechanisms. Neutrophil trogoptosis critically requires CD11b/CD18-dependent conjugate formation and the signaling downstream of the Fc receptors on neutrophils, including the activity of tyrosine kinase (Syk), phopshoinositol-3-kinase (PI3K), myosin light chain kinase (MLCK), and intracellular Ca^2+^. Furthermore, destruction of antibody-opsonized tumor cells by neutrophils is enhanced by blocking the CD47-SIRPα checkpoint^102^ (**Figure 3C**).

Neutrophils might appear to play dual or even opposite roles in tumor immunity and interactions. However, the underlying mechanisms responsible for this discrepancy are the tumor stage and the tumor context, which are essential factors affecting the roles of neutrophil functions in enhancing or suppressing cancer progression. The production of cytokines, chemokines, and growth factors found in the TME in different tumor stages may also contribute to the properties of neutrophils.

## Neutrophils in tumor metastasis

Neutrophils are actively involved in, and have been reported to mediate, the following steps of cancer progression: cancer cell dissemination from the primary tumor, intravasation into the circulation or the lymphatic vascular system, prolongation of tumor cell survival in the blood circulation, extravasation into distant tissues and organs, and outgrowth of metastasis. Although previous studies have mostly supported the pro-metastatic role of neutrophils, an opposing role of anti-metastatic properties of neutrophils has also been reported^44^.

### The pro-metastatic role of neutrophils

**Figure 4:**
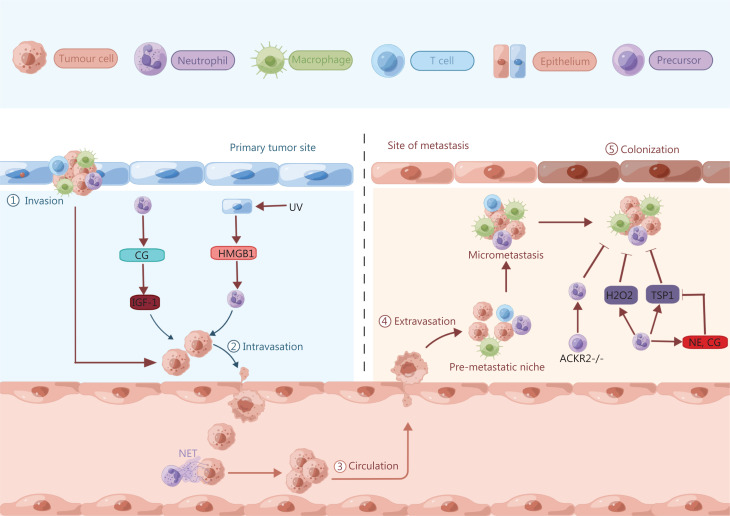
Neutrophils are involved in metastatic progression. Neutrophils are actively involved in the following steps of cancer metastasis: dissociation from the primary tumor, intravasation into the circulation, extravasation into distant tissues and organs, and outgrowth of metastasis. In a melanoma mouse model, UV-damaged epidermal keratinocytes release HMGB1, thus recruiting and activating neutrophils in primary tumors, and subsequently promoting tumor angiogenesis and the ability of melanoma cells to migrate toward endothelial cells. The neutrophil-derived serine protease CG promotes MCF-7 cell migration *via* the activation of IGF-1, and IGF-1 then enhances E-cadherin-mediated intercellular adhesion and tumor cell aggregation, which in turn facilitate tumor cell intravasation into blood vessels. In a murine model of infection using cecal ligation and puncture, NETs support metastasis through sequestering disseminating cancer cells in the circulation and facilitating their seeding at distant anatomical sites. In a breast cancer model, tumor-entrained neutrophils produce H_2_O_2_, thereby preventing metastatic seeding in the lungs. In an experimental 4T1 metastasis model, neutrophils hinder the formation of metastasis through the upregulation of TSP1, in a manner systemically induced by tumor-secreted prosaposin. Neutrophils degranulate azurophilic granules, which release the serine proteases NE and CG, thus resulting in the proteolytic destruction of TSP1. ACKR2 deletion in neutrophil precursors enhances the expression of inflammatory chemokine receptors and mobilization, thus increasing neutrophils’ anti-metastatic activity in 4T1 breast cancer and B16F10 melanoma metastasis models.

The initiation of the cancer metastatic cascade, e.g., dissemination from the primary tumor, intravasation, and priming of the premetastatic niche, has been overlooked in experimental metastasis models^44^. As described earlier, neutrophils are involved in promoting angiogenesis *via* secretion of MMP9, which degrades the extracellular matrix. This process provides more routes for cancer cells to disseminate from the primary site into the circulation to establish a distal seeding site^44^. Neutrophils also guide cancer cells to endothelial cells and facilitate their intravasation into the bloodstream. In a melanoma mouse model, UV-induced epithelial damage has been found to upregulate the level of high mobility group box 1 (HMGB1), thus leading to the recruitment of TLR4^+^ neutrophils to primary tumor sites, and subsequent expansion of the tumor cells toward blood vessels and entry into the blood stream^112,113^. The neutrophil-derived serine protease Cathepsin G (CG) promotes the migration of tumor cells *via* insulin-like growth factor 1 (IGF-1) activation. IGF-1 then enhances E-cadherin-mediated intercellular adhesion and tumor cell aggregation, thereby further facilitating tumor cell intravasation into blood vessels^114^ (**Figure 4**). Collectively, these findings suggest that neutrophils trigger cancer cell interactions with endothelial cells around the primary tumor site, thus resulting in the intravasation of cancer cells into the circulation and the formation of metastasis.

Interestingly, as an abscopal result of tumor cell proliferation at the primary site, neutrophils can accumulate in distant organs before the arrival of disseminated cancer cells and form a region termed the premetastatic niche. For example, in a lung metastatic MMTV-polyoma middle T antigen (PyMT) mammary tumor mouse model, leukotrienes derived from neutrophils promote tumor cell colonization in distant organs through the selective expansion of the sub-pool of cancer cells with high potential for tumorigenesis. Genetic deletion or pharmacologic inhibition of the enzyme arachidonate 5-lipoxygenase (Alox5), which generates leukotrienes, suppresses the pro-metastatic activity of neutrophils and consequently decreases cancer metastasis. Additionally, metastatic progression is retarded in a MMTV-PyMT^+^-Ela2-Cre-DTA^+^ mouse model when lung neutrophils are specifically deleted in tumor-bearing mice^115^.

Increasing evidence demonstrates that NETs also support metastasis by sequestering disseminating cancer cells in the circulation and assisting in their settling in distant tissues^116–121^. In a metastatic breast cancer model, metastasis-supporting NETs proliferate around disseminated cancer cells in the lungs, thereby stimulating cancer cell migration and invasion in a feedback loop. Accordingly, intraperitoneal injection of DNase I-coated nanoparticles significantly decrease lung metastases in mice^119^. In addition, during ovarian cancer, the construction of NETs in the pre-metastatic niche plays an essential role in tumor cell seeding in the omentum. Genetic or pharmacologic inhibition of peptidylarginine deiminase 4 (PAD4), an enzyme critical for NET formation, decreases omental metastasis in mice^122^. Recent studies have also shown that NET-affiliated DNA interacts with the receptor coiled-coil domain containing protein 25 (CCDC25) on tumor cells, thus promoting the adhesion, motility, and growth of metastatic cancer cells in the liver^123,124^. Therefore, studies have provided new understanding of the molecular interactions between NETs and tumor cells.

### The antimetastatic role of neutrophils

In contrast, reports have also shown that neutrophil accumulation and activation in the pre-metastatic niche decrease metastasis. Tumor-derived G-CSF and CCL2 induce the recruitment and activation of neutrophils in pre-metastatic lungs. Subsequently, these tumor-entrained neutrophils prevent metastatic seeding in the lungs through mediating H_2_O_2_ dependent killing of tumor cells^125^.

In addition to release of H_2_O_2_, neutrophils have been found to inhibit the formation of metastasis through upregulation of thrombospondin 1 (TSP1)^126^ in an experimental metastasis model^100^. Tumor cells secrete prosaposin systemically, which in turn induces expression of the anti-tumorigenic factor TSP1 in recruited neutrophils, thereby resulting in a metastasis-refractory microenvironment^126^. Furthermore, neutrophils degranulate azurophilic granules, which deliver the serine proteases NE and CG, thus leading to TSP1 proteolysis. Genetic depletion of these serine proteases protects TSP1 from degradation and inhibits lung metastasis^127^ (**Figure 4**).

In stark contrast to the findings from earlier studies, the recruitment and activation of neutrophils in the metastatic niche have been found to be essential for decreasing metastasis because of the corresponding killing of cancer cells^128,129^. Genetic deficiency in the atypical chemokine receptor 2 (ACKR2) promotes the expression of chemokine receptors on hematopoietic progenitors such as CCR1, CCR2, and CCR5. Activated anti-metastatic cytotoxic neutrophils are released from the BM in response to this increased expression, and they travel toward tumor cells with an anti-metastatic profile^129^. Accordingly, metastasis caused by transplantation with 4T1 breast cancer cell lines or by intravenous injection with B16F10 melanoma cell lines has been found to decrease in ACKR2 deficient mice (**Figure 4**). Thus, inactivating ACKR2 expression activates neutrophils’ anti-metastatic properties, and this method may serve as an innovative means of developing myeloid checkpoint therapies.

## Conclusion

Neutrophils exert dual, apparently opposite, effects on tumor growth and metastasis. Neutrophils mediate DNA instability, and tumor cell proliferation and metastasis, and inhibit the innate and adaptive lymphocyte-mediated anti-tumor immune responses. In contrast, neutrophils engage in direct and indirect crosstalk with other immune cells, thus resulting in killing of tumor cells. These contradictory roles have been ascribed to the heterogeneity of neutrophils in distinct tumor contexts and interactions, thus leading to the differentiation of a variety of neutrophil subpopulations with opposing functions. Hence, a detailed examination of neutrophil heterogeneity in different tumor types and stages is necessary to enable inclusive and comprehensive classification of neutrophils, and to define the different clusters with specific gene signatures in cancer.

Neutrophil heterogeneity in cancer biology has attracted the attention of numerous researchers. Conventionally, neutrophil subpopulations have been divided on the basis of gradient centrifugation methods and polarization states. Single-cell resolution cell profiling has enabled definition of the transcription and protein profiles of different neutrophil subsets and supported a detailed elucidation of neutrophil heterogeneity in tumor conditions. However, different neutrophil subpopulations have diverse properties in different tumor tissues and contexts, and the definitions of neutrophil clusters vary across studies. Moreover, agreement is lacking regarding subpopulation surface markers, thus limiting knowledge regarding neutrophil heterogeneity in tumor conditions. Therefore, correlation analysis and experimental studies are required to reveal the neutrophils in tumor conditions in detail.

Under physiological conditions, neutrophil differentiation from undifferentiated proNeu to terminally differentiated mNeu in the BM progresses linearly. Neutrophil heterogeneity in steady state was believed to be persistent, on the basis of the existence of G5a and G5b. However, tumor-derived TGFβ directs neutrophils toward N2 with pro-tumoral properties, and TGFβ blockade converts N2 neutrophils to an antitumor N1 phenotype, thus indicating that neutrophil heterogeneity under tumor conditions is transient and plastic. The process of neutrophil development in the BM is tightly controlled by specific transcription factors. For instance, the CCAAT/enhancer-binding proteins (C/EBPs), including C/EBPα, C/EBPβ, and C/EBPε, have important roles in regulating neutrophil differentiation. C/EBPα drives CMP differentiation, and its disruption impairs GMP production, thus resulting in complete loss of mature neutrophils. C/EBPβ is highly expressed in G4 and G5a–G5c, and may play an essential role in mature neutrophils. C/EBPε is involved in GMP differentiation to myelocytes and has been suggested to play an important role in the terminal step of the neutrophil development. In addition, growth factor independent-1 (Gfi-1) plays an essential role in facilitating neutrophil differentiation^27^. Neutrophil heterogeneity under tumoral influence is modulated by differential transcription factor expression, which can be induced by tissue and tumor stimuli factors. In the tumor environment, tumor-derived TGFβ dictates the TAN phenotype and skews TAN differentiation toward the N2 pro-tumorigenic phenotype. In addition, the transition from HDNs to LDNs can also be driven by TGFβ. Tumor-derived TGFβ regulates the polarization and transition of neutrophils by activating the transcription factor SMAD. In contrast to TGFβ, tumor-derived IFN-γ and GM-SCF synergistically promote the differentiation of immature neutrophils into a subpopulation of APC-like hybrid neutrophils with anti-tumoral properties by downregulating the expression of transcription factors such as Ikaros^105^. This evidence indicates that neutrophil heterogeneity under physiological and pathological states may be a fixed programmed response potentially mediated by local or tumor-derived factors.

Although enormous achievements have elucidated the biology, functions, and heterogeneity of neutrophils, several questions remain to be addressed regarding the roles of neutrophils in tumors. Currently, processes revealing the functions of different neutrophil subsets in cancers are complex because the initial heterogeneity of neutrophils, compounded by the transformations and alterations in the TME, creates a complex landscape of neutrophil expression. Thus, targeting neutrophils to prevent tumor progression remains an appealing direction but has not yet been definitively achieved. Most researchers have concentrated on understanding the immunosuppressive properties or the protumor functions of neutrophils. Consequently, the anti-cancer functions of neutrophils are less understood, and more research is required to enable medical applications.

Another layer of complexity in this field is that similarities and differences exist in the results obtained from mouse or human origin tumor models, which are indeed distinct and have semi-conserved neutrophil properties. Although development of other higher animal models has been attempted, rodents remain the most common and feasible animal models used to study cancer biology.

Finally, the dual roles played by neutrophils in cancer are so profound that even neutrophil proteins such as NE and MPO can directly exert and assist in pro- or anti-tumor functions where the duality mirrors neutrophils themselves. Neutrophil death can affect tumor dynamics as well: on the one hand, NETs can directly nurture tumors and suppress immune cells; on the other hand, neutrophil apoptosis has anti-tumor properties by facilitating immune signaling. Therefore, when properly activated and regulated, neutrophils can initiate cytotoxicity toward tumor cells and unleash their anti-tumor potential, thus potentially enabling the development of new therapeutic strategies targeting cancer.
